# Ethnic Differences in Risk Factors for Obesity among Adults in California, the United States

**DOI:** 10.1155/2017/2427483

**Published:** 2017-03-02

**Authors:** Liang Wang, Jodi Southerland, Kesheng Wang, Beth A. Bailey, Arsham Alamian, Marc A. Stevens, Youfa Wang

**Affiliations:** ^1^Department of Biostatistics and Epidemiology, College of Public Health, East Tennessee State University, Johnson City, TN, USA; ^2^Department of Community and Behavioral Health, College of Public Health, East Tennessee State University, Johnson City, TN, USA; ^3^Department of Family Medicine, Quillen College of Medicine, East Tennessee State University, Johnson City, TN, USA; ^4^Fisher Institute of Health and Well-Being, Systems-Oriented Global Childhood Obesity Intervention Program, Department of Nutrition and Health Sciences, College of Health, Ball State University, Muncie, IN, USA

## Abstract

Little attention has been given to differences in obesity risk factors by racial/ethnic groups. Using data from the 2011-2012 California Health Interview Survey, we examined differences in risk factors for obesity among Whites, Latinos, Asians, and African Americans among 42,935 adults (24.8% obese). Estimates were weighted to ensure an unbiased representation of the Californian population. Multiple logistic and linear regression analyses were used to examine the differences in risk factors for obesity. Large ethnic disparities were found in obesity prevalence: Whites (22.0%), Latinos (33.6%), African Americans (36.1%), and Asians (9.8%). Differences in risk factors for obesity were also observed: Whites (gender, age, physical activity, smoking, arthritis, and diabetes medicine intake), Latinos (age, arthritis, and diabetes medicine intake), Asians (age, binge drinking, arthritis, and diabetes medicine intake), and African Americans (gender, physical activity, smoking, binge drinking, and diabetes medicine intake). Females were more likely to be obese among African Americans (odds ratio (OR) = 1.43, 95% confidence interval (CI) = 1.05–1.94), but less likely among Whites (OR = 0.80, 95% CI = 0.74–0.87). Race/ethnicity should be considered in developing obesity prevention strategies.

## 1. Introduction

The obesity epidemic in the United States (U.S.) has become a public health crisis [1–6]. Data from the 2011-2012 U.S. national survey showed that the prevalence of adult obesity was 34.9% and varied by ethnicity (e.g., 10.8% for non-Hispanic Asians, 32.6% for non-Hispanic Whites, 42.5% for Hispanics, and 47.8% for non-Hispanic Blacks) [3]. With the exception of Asians, the prevalence of grade 2 obesity (body mass index [BMI] ≥ 35) and grade 3 obesity (BMI ≥ 40) was significantly higher among racial/ethnic minorities than in Whites, particularly among females [3]. Numerous cross-sectional and longitudinal studies have identified similar trends [4–6]. National health objectives call for the elimination of health disparities and achievement of health equity [7]. Racial/ethnic minorities currently comprise 36.3% of the U.S. population and are expected to increase to more than 50% of the total population by 2050 [8, 9]. Disproportionate increases in the prevalence of obesity have led to persistent disparities, particularly among non-Whites [6].

Among adults in California, 35.0% were overweight and 24.7% were obese in 2014 [10]. An earlier report showed racial/ethnic differences in average BMI in Californian adults for both sexes, with larger difference in females [11]. Limited studies have reported the recent prevalence of obesity among Californian adults by demographics. To date, most research in adult obesity trends has focused on differences in the prevalence of obesity by individual, socioeconomic, or neighborhood characteristics [12–15], with less attention given to differences in obesity risk factors by racial/ethnic groups.

This study aimed to examine obesity-related risk factors among four major ethnic groups (Whites, Latinos, Asians, and African Americans [AAs]) in California using data from the 2011-2012 California Health Interview Survey (CHIS). This information can be used to develop targeted obesity prevention interventions and help eliminate health disparities over time.

## 2. Methods

### 2.1. Data and Sample Selection

The CHIS is a collaborative study of the University of California, Los Angeles Center for Health Policy Research, the California Department of Health Services, and the Public Health Institute. As one of the largest health surveys in the nation, this population-based random-digit dial telephone survey has been conducted every other year in California since 2001. The survey examines public health and healthcare access issues in the state. The 2011-2012 CHIS is the sixth data collection cycle for this survey since 2001. Among the randomly selected households, one adult who was 18 years or older was then randomly selected as the respondent. Details about the sampling design and methodology can be found elsewhere [16]. Procedures for data collection and analysis were approved by the Institutional Review Boards (IRBs) at the participant universities and agencies. The current study was approved by the IRB of the authors' university.

Due to small sample sizes for Pacific Islander (*n* = 73, 0.17%), American Indian/Alaskan native (*n* = 636, 1.48%), and other single/multiple racial groups (*n* = 3,065; 7.14%), we combined them as “other.” In the current study, a total of 42,935 adults were included in the analysis, including 26,376 (61.43%) Whites, 6,453 (15.03%) Latinos, 4,253 (9.91%) Asians, 2,079 (4.84%) AAs, and 3,774 (8.79%) adults classified as “other.”

## 3. Measures

### 3.1. Dependent Variable

Obesity was defined as a BMI of 30 kg/m^2^ or more. BMI was calculated from self-reported height and weight.

### 3.2. Independent Variables

Respondents' race was categorized as White, Latino, Asian, AA, and other. Gender was dichotomized to male or female. Respondents' age at the time of the interview was categorized into three levels: 18–44 years, 45–64 years, and 65 years or older. In addition, respondents were asked about employment status, for example, (i) “full-time employment (>21 hours/week),” (ii) “part-time employment (0–20 hours/week),” (iii) “employed and not at work,” (iv) “unemployed and looking for work,” and (v) “unemployed and not looking for work.” In the present study, employment status was dichotomized to yes (i–iii) or no (iv, v). For physical activity, respondents answered “yes” or “no” to the question “Have you walked at least 10 minutes for leisure in the past 7 days?” Respondents were also asked about smoking habits. Responses included “currently smokes,” “quit smoking,” and “never smoked regularly.” In the present study, smoking status was defined as “never,” “current,” or “past.” Binge drinking for males was defined as those who had five or more drinks on at least one occasion in the past month while female binge drinkers were those that had four or more drinks. Frequent binge drinking was defined as engaging in binge drinking daily or weekly, monthly, or more than monthly but less than weekly in the past year. Infrequent binge drinking was defined as engaging in binge drinking once in the past year, more than once but less than monthly in the past year. For arthritis status, respondents answered “yes” or “no” to the question “Has a doctor ever told have arthritis, gout, or lupus?” With regard to diabetes medicine intake, respondents answered “yes” or “no” to the question “Are you currently taking diabetes pills to lower blood sugar?” Furthermore, serious psychological distress (SPD) was determined using the Kessler psychological distress scale (K6) [17], which comprises six questions asking how often during the past 30 days a person felt “so sad that nothing could cheer them up,” “nervous,” “restless,” “hopeless,” “worthless,” or that “everything was an effort.” SPD is a nonspecific measure of psychological distress that has been psychometrically validated and can detect community DSM-IV cases from noncases. Responses are scored from 0 (none of time) to 4 (all the time) and summed to produce a total score. A score of 13 or above was defined as having SPD [17].

### 3.3. Statistical Analysis

Estimates from the CHIS sample were weighted to ensure an unbiased representation of the Californian population. SAS PROC SURVEYLOGISTIC procedure was used to estimate odds ratios (ORs) and 95% confidence intervals (CIs) for the association between independent variables and the prevalence of obesity. SAS PROC SURVEYREG procedure was used to estimate regression coefficients and standard errors for the association between independent variables and BMI. Multiple logistic and linear regression analyses were used to examine the association between all the independent variables and obesity and BMI. Race was found to have significant interactions with gender, age, physical activity, binge drinking, and diabetes medicine intake (all *P*-interaction < 0.05). Multiple logistic and linear regression analyses were then used to examine the differences in risk factors for obesity and BMI in Whites, Latinos, Asians, and AAs, respectively. All analyses were performed using SAS statistical software, version 9.2 (SAS Institute, Cary, NC, USA).

## 4. Results

Overall, the prevalence of obesity was 24.8% among Californian adults. Compared to Whites (22.0%), Latinos (33.6%) and AAs (36.1%) had a higher prevalence of obesity while Asians had a lower prevalence (9.8%). Individuals who were male, between the ages of 45–64 years, and unemployed had a higher prevalence of obesity than their counterparts. Other risk factors for obesity included lack of physical activity, past smoking, arthritis, use of diabetes medicine, and presence of SPD (Table 1).

**Figure 1:**
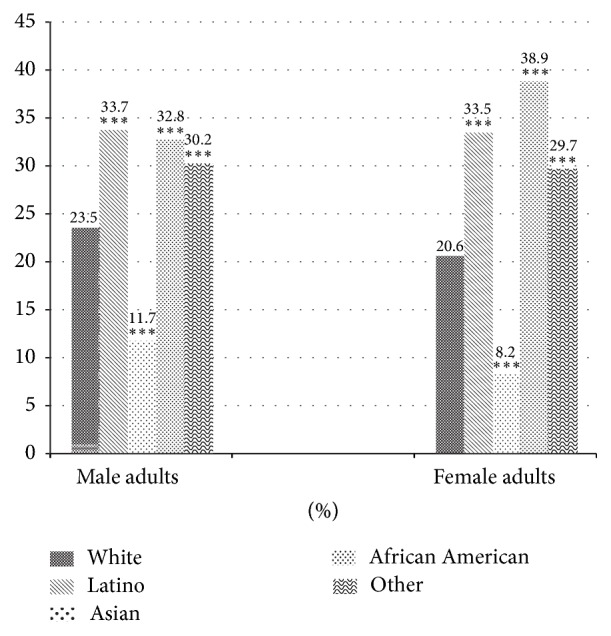
The prevalence (%) of obesity of adults in California in the United States, 2011-2012, by ethnicity and gender. ^*∗∗∗*^*P* < 0.001 versus Whites.* Notes*. *P* < 0.001 for all ethnic groups versus Asians for both sexes. Not statistically significant (NS) for Latinos versus AAs, NS for the others versus AAs among men; *P* < 0.01 for Latinos versus AAs and *P* < 0.001 for the others versus AAs among women. NS for Latinos versus the others among men; *P* < 0.01 for Latinos versus the others among women.

Figure 1 shows the prevalence of obesity by ethnicity and gender. Asian women had the lowest prevalence of obesity, whereas AA women had the highest. Gender disparity in obesity prevalence appeared to be largest in AAs. Among men, ethnic disparities in obesity prevalence were largest between Latino and Asians. Among women, disparities were largest between AAs and Asians. Overall, these differences were greater among women than among men.

Multivariate regression analyses of the associations between all potential risk factors and obesity/BMI are shown in Table 2. Multiple logistic regression analyses showed that compared to Whites, Latinos (OR = 1.94, 95% CI = 1.76–2.15) and AAs (OR = 1.96, 95% CI = 1.66–2.31) had an increased likelihood of being obese while Asians (OR = 0.41, 95% CI = 0.34–0.50) had a lower likelihood of being obese. Other risk factors for obesity included being male, 45–64 years of age, unemployment, lack of physical activity, past smoking, arthritis, and diabetes medicine intake (all *P* < 0.001). Multiple linear regression analyses showed similar results for risk factors of BMI.

Table 3 shows the risk factors associated with obesity for each ethnic group after adjusting for covariates. Multiple logistic regression analyses showed that six risk factors (gender, age, physical activity, smoking, arthritis, and diabetes medicine intake) in Whites, three risk factors (age, arthritis, and diabetes medicine intake) in Latinos, four risk factors (age, binge drinking, arthritis, and diabetes medicine intake) in Asians, and five risk factors (gender, physical activity, smoking, binge drinking, and diabetes medicine intake) in AAs were associated with obesity, respectively.

The estimated ORs varied markedly across these ethnic groups. Females were more likely to be obese for AAs (OR = 1.43, 95% CI = 1.05–1.94), but less likely to be obese for Whites (OR = 0.80, 95% CI = 0.74–0.87); gender was not associated with obesity among Latinos and Asians. Whites and Asians who took diabetes medicine were more likely than Latinos and AAs to be obese (OR (95% CI): 3.74 (3.14–4.45) and 3.25 (2.14–4.96) versus 2.30 (1.79–2.95) and 2.24 (1.41–3.57)). The magnitude of differences in the association of risk factors with obesity also varied by ethnic group. The largest difference of OR was only 0.02 for the association of physical activity with obesity between Whites and AAs but was 1.17 for the association of arthritis with obesity between Asians and Whites.

Table 4 shows risk factors of BMI in different ethnic groups using multiple linear regression. Risk factors of BMI were the same as that of obesity for Whites and Latinos but varied for Asians and AAs. Five risk factors (gender, age, binge drinking, arthritis, and diabetes medicine intake) in Asians and six risk factors (age, physical activity, smoking, binge drinking, arthritis, and diabetes medicine intake) in AAs were associated with BMI, respectively. Specially, being female was associated with lower BMI for Whites and Asians (*β* = −1.20, −1.59, respectively, both *P* < 0.0001) and was not associated with BMI for Latinos and AAs. Whites and AAs with diabetes medicine intake had higher BMI than Latinos and Asians (*β* = 4.42 and 3.40 versus 2.01 and 2.12, respectively, all *P* < 0.0001). The magnitude of differences in the association between BMI and risk factors also varied by ethnic group. The largest difference of *β* was 0.39 for the association of gender with BMI between Whites and Asians but was 2.14 for the association of diabetes medicine intake with BMI between Whites and Latinos.

## 5. Discussion

This study sheds important light on ethnic differences in risk factors associated with obesity. This is one of the first studies to examine variation in risk factors for obesity prevalence across ethnic groups using a large representative sample in California, where large ethnic differences exist in obesity rates. We observed several key findings. First, we found considerable ethnic disparity in adult obesity prevalence: AAs (36.1%), Latinos (33.6%), Whites (22.0%), and Asians (9.8%). The prevalence in AAs was three times larger than that in Asians. The difference in females was even greater; for example, the prevalence in AAs was about 5 times that in Asians (38.9% versus 8.2%). A key strength of our study is that it had an adequate sample of Asians, while other national surveys, for example, National Health and Nutrition Examination Survey, do not include adequate numbers of Asians. It should be noted that several studies have recommended lowering the conventional BMI cutoff points for Asians by 2 to 3 points [18–20]. In 2000, the World Health Organization's (WHO) International Association for the Study of Obesity and the International Obesity Task Force published a report recommending that BMI cutoff of ≥23 and ≥25 for classifying overweight and obese for some Asian groups, respectively [20]. The WHO Expert Consultation have reviewed several publications on this issue and determined that the associations for health risk, BMI, and body fat percentage differ between European and Asian populations. The research shows risk for BMI of 22–25 kg/m^2^ and high risk for ≥26 kg/m^2^ for Asian populations [19]. Another study examined the relationship between BMI and mortality among Asians and showed that high BMI was significantly associated with increased risk of mortality among Asian Americans. The study also reported no significant increase in mortality for Asian Americans with a BMI between 20 and 25. The study is against lowering the cutoff points of overweight and obesity for Asians [21]. Given the apparent lack of consensus on use of lower obesity BMI cutoff points for Asians, we defined obesity as BMI ≥ 30, which may be indicative of the lower obesity percentage for Asians but may not necessarily mean that they are at lower health risk related to weight.

Another key finding is the considerable ethnic variation in the risk factors for obesity.

Only three (age, arthritis, and diabetes medicine intake), four (age, binge drinking, arthritis, and diabetes medicine intake), and five (gender, physical activity, smoking, binge drinking, and diabetes medicine intake) of the nine factors we examined were associated with obesity in Latinos, Asians, and AAs, respectively, while six (gender, age, physical activity, smoking, arthritis, and diabetes medicine intake) of the factors were associated with obesity in Whites. Further, although obesity prevalence was actually higher in AAs and Latinos, risk factors in the present study were more strongly associated with obesity in Whites. These disparate observations suggest that other underlying biological, sociocultural, and environmental mechanisms not accounted for in the present study may have contributed to the obesity prevalence in ethnic minorities. For example, the literature suggests that social context and cultural factors are possible explanations for obesity disparities [22, 23]. Other studies suggest that since the majority of health disparities in adulthood have their etiological origin in childhood [24], it is important to examine factors linked to an individual's childhood such as maternal socioeconomic position [23]. More recent findings suggest, however, that the effect of socioeconomic status on obesity may be diminishing due to the increasing rates of obesity across ethnic, geographic, and community factors [25, 26]. It is important to note that employment status (used as a proxy for socioeconomic status) was not associated with obesity in any ethnic groups.

In this study, age, arthritis, and diabetes medicine intake were the most consistently identified risk factors for higher BMI and obesity. In some cases, age (e.g., among participants who were 65+ years) played a protective role. However, middle age (i.e., 45–64 years) was associated with increasing BMI among all but one ethnic group. Arthritis was positively associated with BMI in all ethnic groups and positively associated with obesity in all ethnic groups except for AAs. Similarly, data from the 2003–2009 Behavioral Risk Factor Surveillance System reported that obesity prevalence in those with arthritis was 54% higher than in nonarthritis patients [27]. Rheumatoid arthritis is an autoimmune disease that causes chronic inflammation affecting multiple synovial joints [28]. This form of arthritis can trigger metabolic alterations [29] that degrade lean tissue especially muscle mass [30]. The most common form of arthritis is osteoarthritis, traditionally affecting more older adults (>60 years) [31, 32]. Combined with an inactive lifestyle, arthritis likely increases accumulation of body fat and body weight leading to reduced muscle mass [33].

On the other hand, research has shown that overweight/obese individuals are more likely to experience osteoarthritis in their knees, hips, and hands [34, 35], suggesting obesity is a risk factor for osteoarthritis, particularly knee osteoarthritis. Studies indicate that obesity increases the weight on a joint causing stress and the breakdown of cartilage that leads to knee and hip osteoarthritis [32, 36]. The cause of hand osteoarthritis has been linked to metabolic factors that are also associated with obesity such as adipocytokines [34]. More longitudinal studies are warranted to determine the potential pathways and interactions between arthritis and obesity.

Diabetes medicine intake was also positively associated with BMI and obesity in all ethnic groups. Adults with type 2 diabetes are more likely to report poorer health-related quality of life including limitations to daily activities thus increasing risk for obesity [37]. Given the increasing prevalence of arthritis (about 22%) and type 2 diabetes (about 10%) among U.S. adults [38, 39], clinicians should screen for chronic conditions to assess risk for obesity, particularly among adults between the ages of 45 and 64 years.

Lack of adequate physical activity is a key contributor of obesity in U.S. adults. Yet in the present study, we found that physical activity protected against obesity only among Whites and AAs. Interestingly, Whites are more likely to meet the federal guidelines for physical activity when compared to Asians and Hispanic/Latino populations (21.3% versus 17.8% and 14.4%, respectively). AAs are slightly less physically active than Asians but more active than Hispanics/Latinos [40]. Lifestyle behavior interventions focused on physical activity habits could result in weight loss in obese adults [5, 41] and reduce risk for obesity.

Our study has some limitations. First, the data are cross-sectional and cannot test causality. Second, data on other potential obesity risk factors such as dietary habits, built environment, and neighborhood level characteristics [22, 42, 43] were not collected. Third, we did not include other known predictors of obesity such as comorbidities, acculturation, and nativity. Moreover, this study utilized self-reported data, which may suffer from report errors and bias. Nevertheless, previous research has shown that such self-reported data can be reliable and are widely used [44]. With regard to BMI, self-report measures are used ubiquitously in large, population-based studies despite the potential for systematic misreporting [45]. Large sample sizes are critical in the epidemiologic examination of various public health issues such as obesity. When using large sample sizes, however, there can be various opportunities for potential bias, including sampling error, measurement error, multiple comparisons errors, aggregation error, and errors associated with the systematic exclusion of information [46]. For this particular study, for example, some participants may have underestimated their BMI. Studies have documented error in self-reported BMI (e.g., over- or underestimates) [45]. While this is a potential limitation, a recent study provides promise. Racial/ethnic minorities (e.g., Blacks, Hispanics, and Native Americans) were found to more accurately report their BMI when compared to Whites [47].

Despite the limitations, the CHIS is the largest state health survey in the nation and one of the few to provide robust samples of many typically underrepresented racial and ethnic groups. Our study adds to the current literature by identifying ethnic variation in risk factors for obesity prevalence using a comprehensive state-level survey for adults. Variations by race underscore the influence of personal characteristics and contextual factors reported in previous studies [13, 14, 22]. There has been growing attention toward eliminating health disparities in the U.S. and toward improving the health of the population as a whole [7]. With the increasing diversity of America's population, it will be important for public health professionals and policy-makers to understand the determinants of obesity risk that are unique to ethnic groups when developing obesity prevention programming, a key target for eliminating health disparities.

## 6. Conclusion

Large disparities in the prevalence of obesity existed across ethnic groups in California. Risk factors for obesity also varied across ethnic groups. Understanding variations in risk factors is important for implementing effective obesity interventions in the increasingly diverse U.S. population. Public health practitioners and policy-makers cannot assume that obesity intervention programs and policies will impact ethnic groups uniformly. Therefore, public health and clinical interventions including communications on reducing obesity risk should be tailored to ethnic groups.

## Figures and Tables

**Table 1:** The prevalence (%) of obesity according to characteristics of participants, the 2011-12 CHIS (*n* = 42,935).

Characteristics	Sample size	Prevalence (%)	95% CI	*P*value^*∗*^
Overall	42,935	24.8	24.2–25.4	
Race				
White	26,376	**22.0**	**21.3–22.7**	**<0.0001**
Latino	6,453	**33.6**	**31.8–35.4**	
Asian	4,253	**9.8**	**8.2–11.4**	
African American	2,079	**36.1**	**32.8–39.4**	
Other	3,774	**29.9**	**27.5–32.4**	
Gender				
Male	17,848	**25.8**	**24.9–26.6**	**<0.0001**
Female	25,087	**23.9**	**23.1–24.7**	
Age in years				
18–44	12,010	**21.8**	**20.8–22.8**	**<0.0001**
45–64	16,810	**30.1**	**28.9–31.3**	
65+	14,115	**23.1**	**22.1–24.1**	
Employment				
No	21,424	**26.3**	**25.2–27.4**	**0.0005**
Yes	21,511	**23.9**	**23.0–24.7**	
Physical activity				
No	15,823	**28.0**	**26.9–29.2**	**<0.0001**
Yes	26,763	**23.0**	**22.1–23.8**	
Smoking status				
Never	25,405	**23.3**	**22.5–24.2**	**<0.0001**
Current	4,977	**25.2**	**23.3–27.1**	
Past	12,553	**28.6**	**27.3–29.8**	
Binge drinking				
Never	32,863	24.9	24.2–25.7	0.378
Infrequent	5,948	25.2	23.5–26.9	
Frequent	4,124	23.4	21.1–25.7	
Arthritis				
No	29,760	**22.5**	**21.8–23.2**	**<0.0001**
Yes	13,175	**33.9**	**32.5–35.3**	
Diabetes medicine intake				
No	38,234	**22.7**	**22.1–23.3**	**<0.0001**
Yes	3,396	**48.6**	**45.8–51.5**	
Serious psychological distress				
No	41,199	**24.4**	**23.9–25.0**	**<0.0001**
Yes	1,515	**35.2**	**30.7–39.7**	

CHIS, California Health Interview Survey; CI, confidence interval.

^*∗*^ *P* value is calculated from *χ*^2^ test.

*Notes*. Sampling weights were used in analysis. Results in bold indicate statistically significant findings.

**Table 2:** Risk factors for obesity and BMI in U.S. adults, the 2011-12 CHIS (*n* = 42,935).

Characteristics	Obesity (based on logistic regression)			BMI (based on linear regression)		
	OR	95% CI	*P* value	*β*	SE	*P* value
Race						
White (ref)						
Latino	**1.94**	**1.76**–**2.15**	<**0.0001**	**2.25**	**0.14**	<**0.0001**
Asian	**0.41**	**0.34**–**0.50**	<**0.0001**	**−1.82**	**0.14**	<**0.0001**
African American	**1.96**	**1.66**–**2.31**	<**0.0001**	**2.10**	**0.22**	<**0.0001**
Other	**1.51**	**1.34**–**1.71**	<**0.0001**	**1.46**	**0.17**	<**0.0001**
Gender						
Male (ref)						
Female	**0.91**	**0.85**–**0.98**	**0.0107**	**−0.74**	**0.08**	<**0.0001**
Age in years						
18–44 years (ref)						
45–64 years	**1.32**	**1.20**–**1.45**	<**0.0001**	**1.09**	**0.13**	<**0.0001**
65+	**0.73**	**0.66**–**0.82**	<**0.0001**	**−0.44**	**0.13**	**0.001**
Employment						
No (ref)						
Yes	0.97	0.88–1.06	0.494	0.01	0.11	0.932
Physical Activity						
No (ref)						
Yes	**0.82**	**0.75**–**0.89**	<**0.0001**	**−0.71**	**0.11**	<**0.0001**
Smoking status						
Never (ref)						
Current	1.00	0.87–1.14	0.956	−0.24	0.13	0.084
Past	**1.19**	**1.09**–**1.29**	<**0.0001**	**0.48**	**0.10**	<**0.0001**
Binge drinking						
No (ref)						
Infrequent	1.05	0.94–1.17	0.427	0.18	0.15	0.233
Frequent	0.99	0.85–1.16	0.906	**−**0.03	0.16	0.850
Arthritis						
No (ref)						
Yes	**1.66**	**1.52**–**1.82**	<**0.0001**	**1.50**	**0.12**	<**0.0001**
Diabetes medicine intake						
No (ref)						
Yes	**2.89**	**2.54**–**3.29**	<**0.0001**	**3.19**	**0.15**	<**0.0001**
Serious psychological distress						
No (ref)						
Yes	1.17	0.95–1.45	0.137	0.41	0.32	0.207

CHIS, California Health Interview Survey; OR, odds ratio; CI, confidence interval.

*Notes*. Sampling weights were used in analysis. Results in bold indicate to be statistically significant.

**Table 3:** Multiple logistic regression analysis of ethnic differences in risk factors for obesity, the 2011-12 CHIS.

Characteristics	Whites		Latinos		Asians		African Americans		Largest difference between the ORs
	OR	95% CI	OR	95% CI	OR	95% CI	OR	95% CI	
Gender									
Male (ref)									
Female	**0.80** ^**∗∗∗**^	**0.74**–**0.87**	1.02	0.85–1.22	0.74	0.52–1.04	**1.43** ^**∗**^	**1.05**–**1.94**	0.63 (African Americans-Whites)
Age in years									
18–44 (ref)									
45–64	**1.39** ^**∗∗∗**^	**1.24**–**1.56**	**1.29** ^**∗∗**^	**1.07**–**1.56**	0.84	0.58–1.22	1.42	0.97–2.08	0.10 (Whites-Latinos)
65+	**0.80** ^**∗∗**^	**0.68**–**0.93**	**0.71** ^**∗**^	**0.53**–**0.96**	**0.53** ^**∗**^	**0.31**–**0.92**	0.65	0.41–1.04	0.27 (Whites-Asians)
Employment									
No (ref)									
Yes	0.96	0.84–1.09	0.95	0.78–1.15	1.24	0.84–1.83	1.08	0.78–1.50	—
Physical Activity									
No (ref)									
Yes	**0.70** ^**∗∗∗**^	**0.64**–**0.77**	0.97	0.81–1.16	1.32	0.95–1.83	**0.68** ^**∗**^	**0.49**–**0.95**	0.02 (Whites-African Americans)
Smoking status									
Never (ref)									
Current	0.96	0.81–1.14	1.07	0.84–1.35	1.06	0.59–1.92	1.17	0.79–1.73	—
Past	**1.17** ^**∗∗**^	**1.05**–**1.30**	1.24	0.99–1.55	0.91	0.61–1.35	**1.57** ^**∗**^	**1.06**–**2.33**	0.40 (African Americans-Whites)
Binge drinking									
No (ref)									
Infrequent	0.92	0.81–1.04	1.11	0.90–1.38	**1.79** ^**∗**^	**1.10**–**2.90**	1.00	0.64–1.56	—
Frequent	0.93	0.77–1.13	0.99	0.74–1.34	1.27	0.64–2.53	**1.73** ^**∗**^	**1.02**–**2.92**	—
Arthritis									
No (ref)									
Yes	**1.60** ^*∗∗∗*^	**1.45**–**1.77**	**1.63** ^*∗∗∗*^	**1.28**–**2.07**	**2.77** ^*∗∗∗*^	**1.86**–**4.13**	1.32	0.92–1.90	1.17 (Asians-Whites)
Diabetes medicine intake									
No (ref)									
Yes	**3.74** ^**∗∗∗**^	**3.14**–**4.45**	**2.30** ^**∗∗∗**^	**1.79**–**2.95**	**3.25** ^**∗∗∗**^	**2.14**–**4.96**	**2.24** ^**∗∗∗**^	**1.41**–**3.57**	1.50 (Whites-African Americans)
Serious psychological distress									
No (ref)									
Yes	1.18	0.88–1.59	1.13	0.80–1.59	1.15	0.53–2.47	1.40	0.66–2.95	—

CHIS, California Health Interview Survey; AOR, adjusted odds ratio; CI, confidence interval.

*Notes*. Sampling weights were used in analysis. Results in bold indicate to be statistically significant.

^*∗*^ *P* < 0.05, ^*∗∗*^*P* < 0.01, and ^*∗∗∗*^*P* < 0.001.

**Table 4:** Multiple linear regression analysis of ethnic differences in risk factors for body mass index (BMI).

Characteristics	Whites	Latinos	Asians	African Americans	Largest difference between the *β*'s
	*β* (SE)	*β* (SE)	*β* (SE)	*β* (SE)	
Gender					
Male (ref)					
Female	**−1.20 (0.09)** ^*∗∗∗*^	0.15 (0.27)	**−1.59 (0.27)** ^*∗∗∗*^	0.61 (0.42)	0.39 (Whites-Asians)
Age in years					
18–44 (ref)					
45–64	**1.17 (0.12)** ^*∗∗∗*^	**1.26 (0.29)** ^*∗∗∗*^	**0.52 (0.26)** ^*∗*^	**0.87 (0.43)** ^*∗*^	0.74 (Latinos-Asians)
65+	**−**0.17 (0.14)	**−0.87 (0.42)** ^*∗*^	0.29 (0.45)	**−2.00 (0.61)** ^*∗∗*^	1.13 (Latinos-African Americans)
Employment					
No (ref)					
Yes	0.11 (0.12)	**−**0.09 (0.32)	0.54 (0.28)	**−**2.29 (0.44)	—
Physical activity					
No (ref)					
Yes	**−0.97 (0.09)** ^*∗∗∗*^	**−**0.46 (0.29)	0.28 (0.23)	**−1.40 (0.40)** ^*∗∗*^	0.43 (Whites-African Americans)
Smoking status					
Never (ref)					
Current	**−**0.28 (0.14)	0.20 (0.34)	0.20 (0.39)	**−**0.78 (0.50)	
Past	**0.41 (0.10)** ^*∗∗∗*^	0.47 (0.26)	0.48 (0.37)	**1.17 (0.52)** ^*∗*^	0.76 (African Americans-Whites)
Binge drinking					
No (ref)					
Infrequent	**−**0.02 (0.14)	0.10 (0.31)	**1.27 (0.41)** ^*∗∗*^	0.63 (0.62)	—
Frequent	**−**0.20 (0.17)	0.001 (0.38)	0.62 (0.51)	**1.82 (0.75)** ^*∗*^	—
Arthritis					
No (ref)					
Yes	**1.37 (0.11)** ^*∗∗∗*^	**1.88 (0.36)** ^*∗∗∗*^	**1.28 (0.31)** ^*∗∗∗*^	**1.51 (0.51)** ^*∗∗*^	0.60 (Latinos-Asians)
Diabetes medicine intake					
No (ref)					
Yes	**4.42 (0.22)** ^*∗∗∗*^	**2.01 (0.33)** ^*∗∗∗*^	**2.12 (0.44)** ^*∗∗∗*^	**3.40 (0.71)** ^*∗∗∗*^	2.41 (Whites-Latinos)
Serious psychological distress					
No (ref)					
Yes	0.30 (0.31)	0.13 (0.47)	−0.27 (0.47)	1.12 (1.24)	—

SE, standard error.

*Notes*. Sampling weights were used in analysis. Results in bold indicate to be statistically significant.

^*∗*^ *P* < 0.05, ^*∗∗*^*P* < 0.01, and ^*∗∗∗*^*P* < 0.001.
